# The Spread of Influenza A(H1N1)pdm09 Virus in Madagascar Described by a Sentinel Surveillance Network

**DOI:** 10.1371/journal.pone.0037067

**Published:** 2012-05-16

**Authors:** Soatiana Rajatonirina, Jean-Michel Heraud, Arnaud Orelle, Laurence Randrianasolo, Norosoa Razanajatovo, Yolande Raoelina Rajaona, Armand Eugène Randrianarivo-Solofoniaina, Fanjasoa Rakotomanana, Vincent Richard

**Affiliations:** 1 Epidemiologic Unit, Institut Pasteur de Madagascar, Antananarivo, Madagascar; 2 Virology Unit, National Influenza Center, Institut Pasteur de Madagascar, Antananarivo, Madagascar; 3 Malagasy Ministry of Health, Antananarivo, Madagascar; National Institute of Allergy and Infectious Diseases, United States of America

## Abstract

**Background:**

The influenza A(H1N1)pdm09 virus has been a challenge for public health surveillance systems in all countries. In Antananarivo, the first imported case was reported on August 12, 2009. This work describes the spread of A(H1N1)pdm09 in Madagascar.

**Methods:**

The diffusion of influenza A(H1N1)pdm09 in Madagascar was explored using notification data from a sentinel network. Clinical data were charted to identify peaks at each sentinel site and virological data was used to confirm viral circulation.

**Results:**

From August 1, 2009 to February 28, 2010, 7,427 patients with influenza-like illness were reported. Most patients were aged 7 to 14 years. Laboratory tests confirmed infection with A(H1N1)pdm09 in 237 (33.2%) of 750 specimens. The incidence of patients differed between regions. By determining the epidemic peaks we traced the diffusion of the epidemic through locations and time in Madagascar. The first peak was detected during the epidemiological week 47-2009 in Antananarivo and the last one occurred in week 07-2010 in Tsiroanomandidy.

**Conclusion:**

Sentinel surveillance data can be used for describing epidemic trends, facilitating the development of interventions at the local level to mitigate disease spread and impact.

## Introduction

The transmission of A(H1N1)pdm09-person to person-is similar to that of other seasonal influenza viruses. Influenza viruses spread rapidly through infected air-borne droplets that are generated by coughing or sneezing. Hands are a major alternative route of viral spread, and can be contaminated “directly”, when droplets land on the hands from an infected person coughing nearby, or “indirectly”, when hands pick up the virus by touching contaminated objects or surfaces. The infectious period for a confirmed case is from one day before the onset of symptoms to seven days after onset [1]–[2].

As concerns community-wide diffusion, influenza epidemics are widespread outbreaks of highly contagious respiratory disease that appear suddenly. Influenza pandemics are characterized by the rapid worldwide spread of a virus to which humans have had no previous exposure.

In infectious disease epidemiology, diffusion is an important concept depicting the dynamics of the spread of a microorganism through time and space [3]. To understand the spread of influenza in a community, it is necessary to study epidemiological and virological conditions and the geographical determinants of influenza during a pandemic [4]. Diverse factors influence the spread of epidemics through human populations, particularly the characteristics of the pathogen responsible for the infection [5], human mobility patterns [6], [7], the sociodemographic structure of the population [4], [8] and intervention measures.

On a population level, influenza spreads both by contagious diffusion (wave-like from one or more central foci) and hierarchical diffusion (movement from larger to smaller towns) [9]. The spread of influenza epidemics is linked more to the rates of movement of people to and from work than to geographical distance or air travel [10]. Also, the virus spreads more rapidly in more densely populated locations.

Recently, the A(H1N1)pdm09 influenza virus swept rapidly across the world after its first detection in humans in April 2009 in Mexico and the US [11], [12]. The A(H1N1)pdm09 pandemic virus is now well-characterized biologically, clinically and epidemiologically [13]. However, little is known about the timing and impact of pandemic influenza in Africa.

The first reported case of A(H1N1)pdm09 influenza to be imported into Madagascar was August 12^th^, 2009. The first laboratory-confirmed cases without travel history were detected on October 8^th^, 2009: three teenagers attending one of the largest schools in Antananarivo [14]. The first wave of A(H1N1)pdm09 peaked in November 2009 in Antananarivo.

Here, we report an analysis of the diffusion pattern of influenza A(H1N1)pdm09 virus by examining all clinical cases reported to the sentinel network. This network allowed the pandemic to be monitored nationwide in real time.

## Results

### Characteristics of the data from sentinel visits

**Figure 1:**
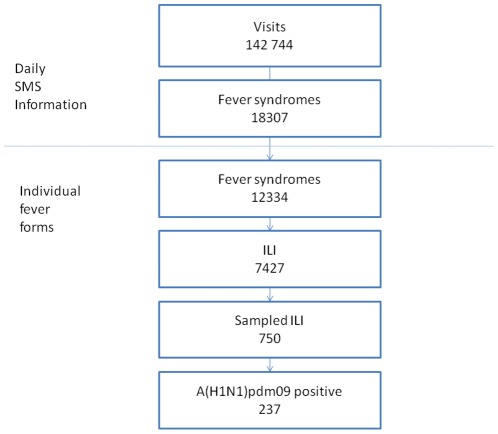
Flow charts of cases from visits to influenza results.

The data, collected on a daily basis between August 1^st^, 2009 and February 28^th^, 2010 from the 24 sentinel centers, corresponded to 142,744 visits (Figure 1); 81.0% of the data were transmitted within 24 h to IPM.

A total of 18,307 cases (12.8%) presented a fever syndrome. A fever-specific form was completed by 12,334 of these patients (67.4%). The sex ratio (male/female) of those with fever syndrome was 0.8; age was available for 12,136 patients (98.4%), and the mean age of these patients was 12.7 years (95%CI: [12.4–12.9]).

Of the patients with a completed fever-specific form, 7,427 cases (60.2%) presented an ILI. The sex ratio (male/female) for those with ILI was 0.9; age was available for 7,316 patients (98.5%), and their mean age was 10.9 years (95%CI: [10.7–11.3]).

Of the patients who presented with ILI, 750 (10.1%) were sampled for laboratory confirmation. Infection with A(H1N1)pdm09 virus was confirmed for 237 (33.2%) patients. The age was available for 233 of these patients (98.3%), and their mean age was 12.6 years (95%CI: [11.1–14.1]).

The age distribution by overall number of visits and febrile syndromes is listed in Table 1. ILI, sampling and laboratory results differed significantly between age groups (p-value<0.01). The proportion of ILI among those with fever syndromes was lower in the population over 25 years old, and the proportion of positive results was higher in the age 5–14year-old group (logistic regression: p-value<0.01).

**Table 1:** Distribution of overall visits, fever-related illnesses, influenza-1-like illnesses, and positive results by age.

Age group	All visits (n = 142,563)		Fever syndromes (n = 12,136)		ILI syndromes (n = 7,316)			Sampled ILI (n = 740)			A(H1N1)pdm positive (n = 233)		
	n	(%)	n	(%)	n	(%)	p	n	(%)	p	n	(%)	P*
<1 year	14,142	(9.9)	1,394	(11.5)	951	(68.2)	<0.01	53	(6.1)	<0.01	11	(20.7)	---
1–4 years	23,774	(16.7)	3,249	(26.8)	2,212	(68.1)	<0.01	223	(11.2)	0.1	52	(23.3)	0.7
5–14 years	24,579	(17.2)	3,485	(28.7)	2,175	(62.4)	<0.01	237	(11.3)	0.1	97	(40.9)	<0.01
15–24 years	26,415	(18.5)	1,944	(16.0)	1,028	(52.8)	<0.01	110	(11.2)	0.2	36	(32.7)	0.1
≥25 years	53,653	(37.6)	2,064	(17.0)	950	(46.0)	---	117	(13.1)	---	37	(31.6)	0.2

* comparison of A(H1N1)pdm positivity by age using logistic regression analysis.

The clinical symptoms are shown in Table 2. The most frequent symptoms presented by A(H1N1)pdm09-positive patients were fever (100%), cough (99.1%), headache (40.1%) and runny nose (38.0%). However, logistic regression analysis of the main symptoms (cough, headache, runny nose, asthenia, sore throat, vomiting, shivering) adjusted for age group only identified a relationship between A(H1N1)pdm09 positivity and runny nose (OR = 1.5; p = 0.03).

**Table 2:** Distribution of symptoms among ILI and confirmed cases of influenza A(H1N1)pdm09 virus infection declared by the sentinel network in Madagascar.

Symptoms	All ILI (n = 7,427)		Sampled ILI (n = 750)		Confirmed Cases (n = 237)	
	n	(%)	n	(%)	n	(%)
GENERAL						
Fever	7,427	(100.0)	750	(100.0)	237	(100.0)
Headache	3,236	(58.1)	281	(47.1)	95	(40.1)
Muscle pain	486	(11.9)	52	(10.7)	20	(8.4)
Join pain	870	(20.5)	68	(13.9)	13	(5.5)
Asthenia	1,047	(14.1)	93	(12.4)	32	(13.5)
Shiver	1,445	(19.5)	130	(17.3)	37	(15.5)
RESPIRATORY						
Cough	6,816	(92.6)	743	(99.1)	235	(99.1)
Sore throat	1,425	(19.2)	96	(12.8)	31	(13.1)
Runny nose	2,283	(30.7)	239	(31.8)	90	(38.0)
Shortness of breath	287	(3.9)	42	(5.2)	5	(2.1)
GASTRO INTESTINAL						
Diarrhea	770	(10.4)	85	(11.3)	22	(9.3)
Vomiting	984	(13.2)	88	(11.7)	31	(13.1)
Nausea	564	(7.6)	55	(7.3)	12	(5.1)
OTHERS						
Retro-orbital pain	81	(2.1)	10	(2.2)	2	(0.8)
Hemorrhagic sign	57	(1.5)	4	(0.8)	2	(0.8)
Skin rash	137	(3.6)	7	(1.5)	0	(0.0)

No case with pneumonia or respiratory failure, requiring ventilation, was reported. Other severe symptoms such as multiple organ failure were not reported.

### Spatio-temporal diffusion patterns: the road of the peaks

**Figure 2:**
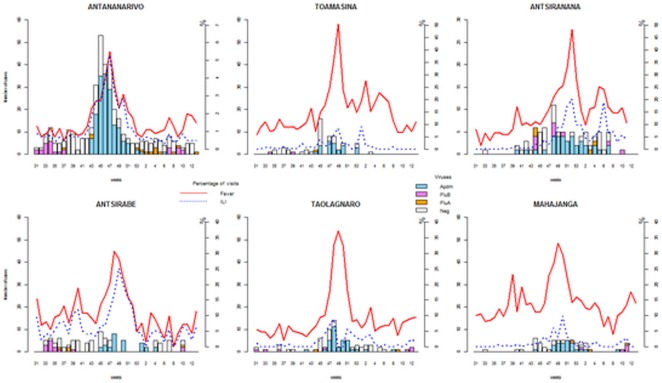
Time series distribution of the fever syndromes, ILI and confirmed influenza cases in the major cities of Madagascar.

**Figure 3:**
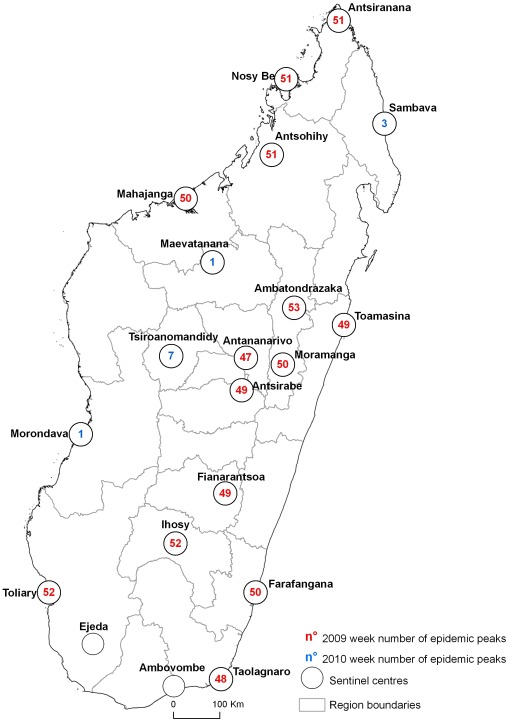
A(H1N1)pdm09 diffusion in Madagascar-the “peaks road.”

Figure 2 shows the progress of the epidemic as assessed from the cases of ILI reported to IPM by the sentinel network. The first peak of ILI (989.3 per thousand visits with fever) was detected during Week 47-2009, in Antananarivo. In this city, cases were confirmed between Week 34 and the first week of 2010 (Table 3). Most cases were confirmed during the first school outbreak which occurred in Week 42-2009. The peak of confirmed cases in Antananarivo occurred in Week 45 (Figure 3) after which systematic detection of all suspected cases ceased.

**Table 3:** Incidence of ILI during each regional peak.

Sentinel Center	Weeks of viral circulation (first-last)	Week of the ILI peak	Incidence of fever syndromes per thousand visits	Incidence of ILI per thousand visits with fever
Antananarivo	34^th^ 2009–01^st^ 2010	47^th^ 2009	62.3	989.3
Taolagnaro	42^nd^ 2009–02^nd^ 2010	48^th^ 2009	326.8	273.8
Toamasina	42^nd^ 2009–50^th^ 2009	49^th^ 2009	503.2	562.3
Antsirabe	44^th^ 2009–02^nd^ 2010	49^th^ 2009	281.0	891.7
Fianarantsoa	48^th^ 2009–52^nd^ 2010	49^th^ 2009	222.2	200.0
Moramanga	48^th^ 2009–52^nd^ 2010	50^th^ 2009	424.2	46.3
Mahajanga	44^th^ 2009–09^th^ 2010	50^th^ 2009	294.5	336.6
Farafangana	46^th^ 2009	50^th^ 2009	215.7	597.4
Antsiranana	43^rd^ 2009–03^rd^ 2010	51^st^ 2009	330.1	543.5
Nosy Be	No swab	51^st^ 2009	461.0	538.5
Antsohihy	36^th^ 2009	51^st^ 2009	391.3	111.1
Ihosy	No swab	52^nd^ 2009	435.9	117.6
Toliara	47^th^ 2009–06^th^ 2010	52^nd^ 2009	38.2	333.3
Ambatondrazaka	No swab	53^rd^ 2009	135.1	600.0
Morondava	52^th^ 2009–01^st^ 2010	01^st^ 2010	124.7	150.9
Maevatanana	49^th^ 2009–08^th^ 2010	01^st^ 2010	221.7	446.9
Sambava	No swab	03^rd^ 2010	271.3	57.9
Tsiroanomandidy	49^th^ 2009–08^th^ 2010	07^th^ 2010	195.1	250.0

The incidence of ILI was also evaluated by region (Table 3). The peaks were observed through the various sentinel centers in Madagascar and occurred from Weeks 47-2009 to 07-2010 (Figure 2).

## Discussion

We studied the epidemiology of the current pandemic of influenza A(H1N1)pdm09 virus in Madagascar, including the temporal and geographic pattern of spread and the clinical characteristics of A(H1N1)pdm09 disease among ILI patients. We characterized the range and chronology of the pandemic in Madagascar with real-time data from a sentinel network.

This is the first description of overall trends of pandemic influenza A(H1N1)pdm09 infection in Madagascar. This country was one of the first countries in Africa to report laboratory-confirmed cases of this new influenza virus: cases of pandemic influenza were detected in Madagascar before mass gatherings occurred. All of the initial sporadic cases identified were imported (from India, Mauritius and Reunion Island). The sentinel surveillance networks which have been operational since 2007 [15] presumably contributed to this because coverage was good in high density population settings; the updated information given to health care professionals in June 2009 during an influenza workshop organized by Institut Pasteur from Madagascar may also have contributed.

The pattern of spread of A(H1N1)pdm09 in Madagascar was dominated by a wave that emanated from the capital, Antananarivo. The early dynamics of this wave might have been associated with a high frequency of international travel, increasing the risks of a major epidemic in the capital city [10].

It was challenging to implement the early phase of the WHO recommendations concerning the epidemic. Thus, epidemiological investigation was crucial to monitor the epidemic better, particularly to identify risk groups and factors that contributed to the development of the epidemic. An understanding of the epidemiology of past pandemics, and in particular the last A(H1N1)pdm09 pandemic, may help health authorities to prepare and implement response programs to subsequent waves and pandemics [16].

Collecting relevant information on pre-existing chronic conditions and complications among hospitalized cases could be valuable to fill the gaps in the existing epidemiological data. However, in Madagascar, like in others developing countries, the level of hospital care is poor, and there is a lack of influenza surveillance in hospitals (e.g. concerning severe cases). This weakness with regard to surveillance, and hospitalization costs for patients, need to be considered and overcome so as strengthen surveillance systems with data from hospitals.

Our report demonstrates that the signs and symptoms in ILI or confirmed cases are similar to those observed in patients with seasonal influenza [16], [17]. We observed that sampling children aged under 1 year old was much less effective than that for other groups. The proportions of patients sampled did not differ between the other age groups, and the positivity rate for A(H1N1)pdm09 was high in patients aged between 4 and 15 years. One characteristic of this pandemic is that it disproportionately affected children and young adults [18]; indeed, a study in the USA reported that 60% of confirmed A(H1N1)pdm09 cases were aged 18 years or younger [19].

We also observed that transmission of the influenza A(H1N1)pdm09 virus during the pandemic in Madagascar, like in other countries, appeared to be inevitable, presumably due to the nature of the infection: the virus spread despite preparations to mitigate the situation, including systematic detection of all suspected cases, social distancing options (such as school closure) and antiviral treatment of all confirmed cases and their contacts during the early stage of the epidemic. However, in Madagascar like in other remote areas, the impact of vaccination or timely oseltamivir use on influenza transmission was probably low because these measures were unavailable throughout the country.

As in other countries [20]–[22], there were regional differences in the trend of the epidemic and the timeline of A(H1N1)pdm09 spread, and the causes are not easily explained. These differences may be associated with the demographic structure or population density of these regions. The region specificity of the pattern requires further examination.

Data from Madagascar, like those from others countries [23], suggest that the A(H1N1)pdm09 virus spreads rapidly through communities once introduced from an affected area. For the specific case of Madagascar Island, the principal spread of infection took approximately 3–4 months. This rapid spread of pandemic influenza infection across the whole country underscores the need for real-time surveillance systems to track viral activity across regions and districts.

In conclusion, the sentinel data allowed description of the pattern of disease activity. Both seasonal and pandemic influenza surveillance, using sentinel data, is informative when combined with laboratory testing. We advocate (i) enhancing the surveillance capacity with the aim of mitigating the course of future epidemics early, (ii) strengthening surveillance efforts, and (iii) promoting information sharing in Africa because the influenza epidemiology on the continent is largely unknown. In addition population-based serological surveys should be performed to generate more accurate estimates of the epidemic's impact.

## Methods

### Subjects

Before the pandemic was declared in Madagascar, the surveillance aimed to identify cases in travelers returning from affected areas to allow prompt implementation of control measures around each case (nasal swab, and antiviral treatment if case was confirmed) and to contain viral spread. Antiviral prophylaxis was recommended for close contacts of confirmed cases, who were asked to quarantine themselves at home.

The case management protocol was updated according to the general dissemination of the virus and the start of transmission in the community; ultimately, the protocol was limited to patients with known risk factors and hospitalized cases.

As described previously [15], the sentinel surveillance network was based on daily declaration by volunteer general practitioners (GPs) throughout the 22 regions of Madagascar. Participating GPs reported daily the total number of consultations and any patients who presented with influenza-like illness (ILI), defined as observed fever (>38°C) and cough or sore throat.

With the emergence of the influenza A(H1N1)pdm09 virus, the following case definitions of suspected and confirmed cases were used:

A suspected case of influenza A(H1N1)pdm09 virus infection was defined as a person with ILI who meets at least one of the following epidemiological criteria of the WHO case definition protocol [24]: (i) returned from a country or region with an epidemic of influenza A(H1N1)pdm09 virus within the last seven days, (ii) was in close contact with a confirmed case within the past seven days, or (iii) handled samples suspected of containing influenza A(H1N1)pdm09 virus in a laboratory or other setting within the past seven days. A confirmed case of influenza A(H1N1)pdm09 virus infection was defined as a person with laboratory confirmation by real-time PCR. For each case, we collected demographic (including age and sex) and clinical (fever, cough) data on a dedicated fever case report form.

### Laboratory confirmation

Nose and throat swabs were collected from all suspected cases during the first step of the spread and on a random basis during community spread. Respiratory specimens were placed in universal transport media (Copan, Italia), and transported at 4°C twice per week from sentinel sites to the National Influenza Center (NIC) at the Institut Pasteur of Madagascar (IPM) for confirmation. Specimens were stored at 4°C before being tested by PCR.

Samples were also tested for other seasonal influenza viruses during the period (Influenza types B and A, and subtypes AH1 seasonal and AH3 seasonal)

The CDC Real-Time RT-PCR Protocol for the detection and characterization of human and swine influenza was used to confirm cases. It includes panels of oligonucleotide primers and dual-labeled hydrolysis (Taqman®) probes for *in vitro* qualitative detection and characterization of human and swine influenza viruses in respiratory specimens [25]. The Ambion Ag-Path One-Step RT-PCR kit was used for this assay.

### Data analysis

Statistical analysis was performed using R version 2.12.0 [26]. Arcview 9.2 was used for mapping.

Descriptive analyses comprised assessing frequency distributions and proportions for each variable category. Fisher's exact test for categorical variables was used for group comparisons, and ANOVA for continuous variables. P values were two-sided.

Logistic regression analysis was performed to measure the association between ILI or laboratory results and each independent variable (symptom, age group). Odds ratios (ORs) and 95% confidence intervals (CIs) were calculated from â coefficients and their standard errors.

### Ethical clearance

The data were collected by routine surveillance and were anonymous; thus, the epidemiologists running the surveillance network had ethical and professional obligations to maximize the benefits of the studies to the participants and society, and minimize potential harm (such as loss of privacy and confidentiality). These risks were remote possibilities due to the steps that were taken to safeguard confidentiality: they included data encryption, written procedures for confidentiality, and appropriate staff training. Malagasy public health authorities can legally collect and receive information for the purposes of preventing and controlling disease, injury, and disability. Hence, no specific ethics approval is required for public health practice activities such as surveillance. Nevertheless, verbal informed consent-as noted on the form by the primary health care staff-was obtained from each patient, or from at least one parent for children, before sampling under the principle of respect for individual freedom.
